# Pyogenic Liver Abscess Caused by Methicillin-Susceptible* Staphylococcus aureus* in a 21-Year-Old Male

**DOI:** 10.1155/2018/9868701

**Published:** 2018-06-19

**Authors:** Samuel Igbinedion, Meher S. Mavuram, Moheb Boktor, John Bienvenu

**Affiliations:** ^1^Department of Internal Medicine, Louisiana State University Health Sciences Center, Shreveport, LA, USA; ^2^Department of Gastroenterology and Hepatology, Louisiana State University Health Sciences Center, Shreveport, LA, USA

## Abstract

Liver abscesses are the most common types of visceral abscesses. Pyogenic liver abscesses, a particular type of liver abscesses, are uncommonly encountered. We present a rare case of pyogenic liver abscess caused by methicillin-susceptible* Staphylococcus aureus* in a young man. A 21-year- old man presented from prison to the hospital with fever, nausea, vomiting, diarrhea, and abdominal pain for five days. Labs were significant for leukocytosis with predominant neutrophilia and elevated liver enzymes. CT abdomen with contrast revealed an 8.4 cm multiloculated right hepatic mass extending to the kidney. Patient was started on broad spectrum antibiotics, given septic presentation. Peripheral blood cultures returned positive for methicillin-susceptible* Staphylococcus aureus* (MSSA). The culture from percutaneous drainage also revealed MSSA. He received a total of four weeks of IV Nafcillin therapy along with drainage of his abscess via percutaneous catheter. Follow-up revealed clinical resolution. This case highlights the importance of obtaining an aspirate from the liver abscess to better guide treatment strategy. Clinicians must consider broadening antibiotic coverage to include gram-positive organisms if the patient presents with severe illness and risk factors for* Staphylococcus aureus* infections.

## 1. Introduction

Liver abscesses are the most common types of visceral abscesses [1]. Liver abscesses are divided into three main categories based on the predisposing condition: iatrogenic, infectious, or malignant. Infectious and iatrogenic abscesses could be pyogenic or parasitic in origin [2]. Pyogenic liver abscesses (PLAs) are very uncommon although recent study has shown increasing incidence in the United States [3]. Pyogenic liver abscess is associated with significant morbidity, mortality, and health care costs [3]. Common predisposing conditions include underlying gastrointestinal malignancy, history of a previous biliary surgery or endoscopy, immunosuppression, and diabetes mellitus [4]. Before the advent of antibiotics, patients presented with fever, abdominal pain localized to the right upper quadrant (RUQ), and, in some severe cases, shock. Since the advent of antibiotics, presentation has become milder and is now commonly seen in older adults. Patients now typically present with symptoms such as malaise, low-grade fever, weight loss, and RUQ pain [4]. This case highlights a rare presentation of pyogenic liver abscess in a young 21-year-old male prisoner with no comorbid condition with the cause identified as secondary to methicillin-susceptible* Staphylococcus aureus*.

## 2. Case Report

A 21-year-old man with no known past medical history presented from prison with abdominal pain in the past five days. He explained that five days prior to presentation, he began experiencing squeezing abdominal pain, rated 4/10 localized to his RUQ with no radiation. He reported associated nausea, vomiting, diarrhea, and intermittent fever. The patient reported that he had initially thought he had the “stomach flu.” He had tried oral rehydration and Tylenol with no improvement in symptoms since onset. He reported continual progression of symptoms with minimal resolution in abdominal pain, episodes of vomiting, or diarrhea. He denied any change in diet, change in eating habits, recent travel, jaundice, tea-colored urine, itching, recent skin, and soft tissue infection. He stated that he was a previous smoker but quit since being incarcerated two years ago. He denied any alcohol or illicit drug use. He denied having any current sexual partner. He also denied any family history of illnesses or malignancy. On physical examination, patient was normotensive, tachycardic, and febrile on presentation with temperature recorded as 103.7F. Patient appeared weak and diaphoretic and had moderate right upper quadrant tenderness with no rebound tenderness or mass palpated and no other pertinent abdominal findings. Examination of the other systems revealed no abnormalities. The patient was admitted for further work-up of his condition given presentation from prison.

**Figure 1:**
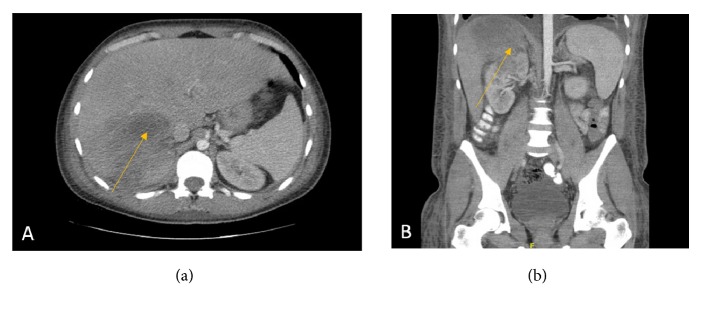
(a) Axial CT image of the abdomen showing a large multiloculated right hepatic mass extending from segment VIII to segment VI (arrow). (b) Coronal image in the same patient showing extension of the hepatic mass into the upper pole of the right kidney.

Initial laboratory results showed normocytic anemia (hemoglobin of 9.8, MCV 81.1), white blood cell count of 15.6 with significant left shift, sodium of 137 mmol/L (reference value 136-145 mmol/L), and potassium of 3.4 mmol/L (3.5-5.1 mmol/L) with normal renal function. Other significant labs on presentation included alanine aminotransferase (ALT) of 30 U/L (12-78 U/L), aspartate aminotransferase (AST) of 43 U/L (15-37 U/L), alkaline phosphatase (ALP) of 240 U/L (45-117 U/L), total bilirubin of 1.5 mg/dL (0.2-1.0 mg/dL), albumin of 1.4 g/dL (3.4-5.0 g/dL), and total protein 5.8 g/dL (6.3-9.0 g/dL). Rapid influenza testing was negative. Urine drug screen resulted as negative. Blood cultures from two peripheral sites were collected. Chest X-ray was significant for bibasilar infiltrates with bilateral pleural effusions noted. Given the clinical condition with concern for intra-abdominal source of infection, a CT scan of the abdomen/pelvis with IV contrast was performed. This showed a large multiloculated right hepatic mass, measuring 8.4cm with soft tissue mass extension into the upper pole of the right kidney (Figure 1), unremarkable findings in the gallbladder, bowel, and appendix.

In the Emergency department, the patient was initially started on aggressive hydration with 0.9% normal saline given initial presentation prior to work-up. Ceftriaxone 1g Intravenous (IV) route and Metronidazole 500mg Intravenous (IV) route were administered in the emergency department. Blood cultures obtained from two peripheral sites resulted as gram-positive cocci with time to positivity about 15 hours. Patient was started on intravenous broad spectrum antibiotics with Vancomycin 1g IV q12 hours and Meropenem 1g IV q8 hours, the initial choice of therapy. Antibiotic sensitivity resulted in day two of admission as MSSA. Antibiotic therapy was narrowed down to Intravenous Nafcillin on day two of admission. Interventional radiology department was consulted for image guided percutaneous drainage of the liver abscess on day one of admission. Two abdominal drains were placed with initial return of 150ml of serosanguinous fluid. Fluid obtained from the CT-guided aspiration was sent to the laboratory for gram stain and culture. The culture obtained from percutaneous catheter drainage resulted as MSSA on day two of admission. The source of MSSA bacteremia was confirmed as pyogenic liver abscess and Nafcillin IV therapy was continued with no change in antibiotic management. Abdominal drains were removed on day six of admission as output remained minimal.

The patient was found to have bilateral pleural effusions. Given significant right sided pleural effusion, thoracentesis was performed on day two of admission with 1.2L of pleural fluid removed. Fluid was not sent for studies. Patient also developed nonoliguric acute kidney injury with creatinine rise from 0.7mg/dl (reference value 0.7-1.3 mg/dL) on admission to 1.7 mg/dL on day four of admission. Work-up was consistent with an intrinsic etiology of the acute kidney injury. The etiology was clinically suspected to be secondary to acute tubular injury due to sepsis. The patient was encouraged to continue per oral intake and intravenous fluids were given. Creatinine trended back to patient's baseline while on Nafcillin IV therapy. Repeat blood cultures obtained on day four of admission were consistently growing MSSA. Transthoracic echocardiogram was performed and it showed no evidence of intracardiac vegetations and no abnormalities. Repeat blood cultures on day six of admission were negative. A mid-line catheter was inserted in the patient's right upper extremity for vascular access on discharge for continued antibiotic therapy. The patient was discharged after 10 days with continued Nafcillin IV therapy for four weeks postdate of negative blood cultures.

**Figure 2:**
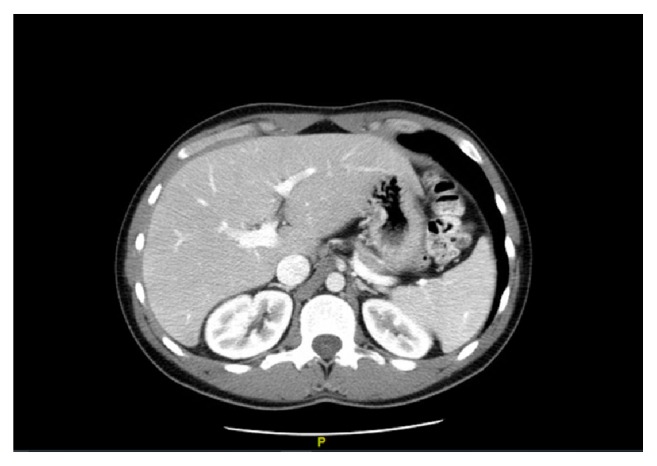
An axial CT imaging of the abdomen showing resolution of pyogenic liver abscess in this patient.

The patient was followed up in the general surgery clinic one month after discharge. At this time, he had completed IV antibiotic therapy. Follow-up revealed stable clinical condition as patient reported resolution of symptoms and return to baseline functional status. Laboratory findings indicated normal renal function. Repeat CT imaging of the abdomen with IV contrast on follow-up visit showed resolution of the pyogenic liver abscess (Figure 2).

## 3. Discussion

Pyogenic liver abscess is an uncommon condition but its incidence, estimated at 8 to 15 per 100,000 population, is now rising in the United States [3, 5]. Biliary tract infections are the most common reported source of liver abscess. Liver abscess can develop from infectious spread or via hematogenous spread in cases of bacteremia arising from skin and soft tissue infection, underlying abdominal disease including appendicitis, and inflammatory bowel disease or rarely from endocarditis [4, 6]. The causative bacteria can gain entry into the liver from adjacent organs or through the portal venous system or the arterial flow. In approximately 40% of cases of pyogenic liver abscess, no obvious source of infection is identified [4].

Most cases of pyogenic liver abscesses are polymicrobial. Commonly isolated organisms include gram-negative bacteria:* Escherichia coli, Klebsiella*,* Proteus, Pseudomonas*, and* Streptococcus *species.* Bacteroides fragilis *and* Fusobacterium necrophorum *are the common anaerobes isolated. Usually,* Staphylococcus aureus *infection is seen only in children or in ill-appearing patients with bacteremia [4].

We illustrate a rare case of pyogenic liver abscess in an otherwise healthy 21-year-old man caused by MSSA. Most cases of patients with liver abscesses reported are among the elderly with the mean age of presentation of 63 years [7]. Common predisposing conditions include underlying gastrointestinal malignancy, history of a previous biliary surgery or endoscopy, immunosuppression, and diabetes mellitus [4]. Our patient had none of these medical conditions which could predispose him to a higher risk of infection.

On presentation of our patient to the hospital, the history of abdominal pain, nausea and vomiting, intermittent fevers, and ill appearance was concerning for an intra-abdominal infection. An ultrasound abdomen was never ordered on initial encounter in the emergency department to better evaluate the patient based on physical findings. Rather, a CT scan of the abdomen/pelvis with contrast initially was ordered for better visualization of the structures in the abdominal cavity. Although ultrasound and CT imaging are the preferred initial imaging modalities of choice in evaluating for liver abscess, obtaining the ultrasound is the inexpensive option [4]. The ultrasound can also guide needle aspiration of the abscess for the clinician.

Infective endocarditis was also considered, given persistent MSSA bacteremia after repeat blood cultures were collected on day four of admission. A transthoracic echocardiogram was performed on our patient with findings negative for valvular regurgitation or vegetation. A transesophageal echocardiogram was not pursued given low suspicion for infective endocarditis as patient did not meet the Duke's modified criteria for diagnosis of infective endocarditis [8]. In our patient, the liver abscess must have developed from the hematogenous spread of the MSSA bacteremia. The underlying infection causing the bacteremia was unable to be identified. Jeong et al. illustrated the need for colonoscopy evaluation in cases of cryptogenic pyogenic liver abscess to detect hidden colonic malignant lesions [9]. Given our patient's age and lack of comorbid condition, the decision was made not to pursue colonoscopy evaluation.

Few cases of pyogenic liver abscess caused by* Staphylococcus aureus* have been reported in the literature, especially in a young healthy individual [10, 11]. Cases caused by* Staphylococcus aureus* have been described in patients who are neonates, elderly, immunocompromised, status postabdominal procedures, or who have gastrointestinal malignancy or biliary disease [4, 10]. Studies have also shown prisoners to be at a higher risk of acquiring* Staphylococcus aureus *infections, particularly MRSA infection [12, 13]. Our case report highlights the case of an incarcerated patient who has no comorbid condition or significant past medical history but presents with a pyogenic liver abscess caused by MSSA. The patient also experienced a remarkable improvement after percutaneous drainage and antibiotic therapy.

Drainage of the abscess is the standard of care for pyogenic liver abscess [4, 14]. This can be achieved percutaneously. Percutaneous needle aspiration (PNA) and percutaneous catheter drainage (PCD) are the methods used to drain the abscess. PCD has been shown to be more effective than PNA in terms of success rate, clinical improvement, and time to achieve 50% reduction in abscess cavity size [14]. Surgical drainage is indicated for abscesses which are difficult to access via percutaneous method [14]. After PCD, our patient achieved resolution of abscess with four-week intravenous therapy. There was no need for further therapy with oral antibiotics. The length of antibiotic therapy after adequate drainage can range from 14 to 42 days depending on clinical response [15]. However, no randomized controlled trials have studied the optimal length of therapy after appropriate drainage.

## 4. Conclusion

Pyogenic liver abscess is a highly fatal condition if untreated [4]. Thus, targeted therapy is ideal in the management of the condition. Drainage of the abscess and antibiotic therapy should be the ideal treatment strategy in pyogenic liver abscess as this has helped in decreasing the mortality rate of this condition. Clinicians must consider broadening antibiotic coverage to include gram-positive organisms if the patient presents with severe illness and risk factors for* Staphylococcus aureus *infections, incarceration in this case [16].
